# The alleviating specific phobias in children trial: Challenges and solutions to implementing a randomized controlled trial in clinical services

**DOI:** 10.3389/frcha.2022.1027083

**Published:** 2022-10-28

**Authors:** Lucy Tindall, Alexander J. Scott, Katie Biggs, Emily Hayward, Jon Wilson, Cindy Cooper, Rebecca Hargate, Barry Wright, Lina Gega

**Affiliations:** ^1^Child Oriented Mental Health Intervention Centre (COMIC), Leeds and York Partnership NHS Foundation Trust, York, United Kingdom; ^2^Department of Health Sciences, University of York, York, United Kingdom; ^3^School of Psychology, Keele University, Keele, United Kingdom; ^4^School of Health and Related Research (ScHARR), University of Sheffield, Sheffield, United Kingdom; ^5^Norfolk and Suffolk NHS Foundation Trust, Norwich, United Kingdom; ^6^Department of Clinical Psychology, University of East Anglia, Norwich, United Kingdom

**Keywords:** specific phobias, Child and Adolescent Mental Health Services (CAMHS), implementation, children, young people

## Abstract

In 2015, The Alleviating Specific Phobias Experienced by Children Trial (ASPECT) was commissioned by the National Institute for Health and Care Research (NIHR) to compare the clinical and cost-effectiveness of multi-session Cognitive Behavioral Therapy (CBT) for specific phobias in children and young people (CYP) (aged 7–16), with a briefer variant called One Session Treatment (OST). From 2016 to 2020, ASPECT recruited *n* = 274 CYP with specific phobias and their families from across England, including 26 Child and Adolescent Mental Health Services (CAMHS) centres, three voluntary sector centers and one University-based wellbeing service. Whilst the trial successfully reached its recruitment target, the challenges experienced in its delivery highlight the difficulties of embedding child and adolescent research into clinical settings and routine practice. Using ASPECT as a case in point, this paper explores these challenges and provides important insights and considerations of potential benefit to others conducting research within the field of child and adolescent mental health.

## Introduction

The Alleviating Specific Phobias Experienced by Children Trial (ASPECT) was commissioned in 2015 by the UK's National Institute for Health and Care Research (NIHR) to compare the clinical and cost-effectiveness of multi-session Cognitive Behavioral Therapy (CBT)—the most common treatment for specific phobias in children and young people (CYP)—with a briefer variant: One Session Treatment (OST) [1, 2]. Between 2016 and 2020, ASPECT recruited 274 participants across 26 Child and Adolescent Mental Health Services (CAMHS) in 13 NHS Trusts, three voluntary agencies, and one University. ASPECT demonstrated that OST yielded similar clinical effects to CBT, is acceptable to CYP, and is cost-saving for services [1, 3]. On this basis, OST should be adopted in routine practice.

Embedding a research study like ASPECT within routine services and care pathways is important so that its findings are grounded in real world settings and are generalizable. This comes with significant challenges. With ASPECT as a case illustration, we describe five key challenges we faced embedding research into routine services and care pathways. In each case, we propose mitigating strategies that can be implemented from the trial's outset. The same challenges apply beyond the duration of the research. Future clinical studies in child and adolescent mental health can benefit from our insights and experiences.

### Challenge 1. Service delivery restructuring and lack of streamlined care pathways

#### CAMHS restructuring

At the inception of any large-scale intervention study in specific patient populations, establishing the routine care pathways to care is key, yet this has become more challenging within CAMHS research. In response to the government papers Future in Mind [4, 5] and the Child Mental Health Green Paper [6] many structural changes within CAMHS have occurred and continue to do so. This has been in parallel with both large reductions in local authority and NHS funding and significant increases in child mental health referral rates across England [7]. Many services have experienced staff reductions and have needed to reorganize resources to help those most in need. Decisions about resource allocation and thresholds for acceptance into CAMHS services are set locally rather than centrally which means that care pathways for many conditions, including phobia, are unclear.

The changes to CAMHS and the frequently unclear care pathways create significant challenges in undertaking research. We were initially confident that embedding ASPECT into a small number of CAMHS settings would be sufficient in meeting our recruitment target. However, due to CAMHS restructuring, phobias were increasingly deemed lower priority, with many CAMHS ceasing to provide phobia support (i.e., CBT). Whilst some services disbanded completely, others stopped accepting phobia referrals; instead passing referrals onto other organizations (e.g., school-based and third sector organizations). Our efforts to increase recruitment by expanding the number of recruiting sites had limited success. Of 77 NHS trusts approached, only 9 (12%) successfully opened for recruitment. Common reasons for non-participation included having low/no phobia referrals (40%) and limited staff capacity for therapy delivery (18%). Expanding recruitment into alternative locations like voluntary agencies and school-based services created further challenges, in the heterogeneity of the sites and the lack of experience in delivering specialist interventions. To tackle this, the research team had to be adaptable and flexible to meet the needs and structures of different services to enable successful trial recruitment.

#### Complex and varied care pathways

The complexity and variation within care pathways for CYP with specific phobia created further challenges when implementing ASPECT. Variation regarding specific phobia related to acceptance thresholds; with some services accepting them routinely, some reporting not receiving them, some not accepting them at all, and others only accepting them only if the case was complex. Consequently, access to phobia treatments varied depending on the locality of the service but also in respect of who should provide treatment and what this should comprise. Despite guidelines by the National Institute for Health and Care Excellence [8] that recommend CBT for phobias, CYP were often referred to non-CBT services as many primary care services and other referrers (i.e., General Practitioners, pediatricians, schools) were uncertain about appropriate and available phobia treatments.

The structural changes to services and lack of streamlined care pathways pose challenges in turn for embedding ASPECT research findings into practice. These challenges include identifying appropriate services, and care pathways within those services, where interventions like OST can be successfully and routinely delivered. This is likely to equally apply to other mental health conditions that are considered lower priority following CAMHS restructuring.

#### Possible solutions

To address these challenges and facilitate research and practice, NICE treatment guidelines need to be made more readily available. For phobias (the focus of ASPECT), there is currently no formal published NICE guidance, instead the identification and management of phobias are covered by the NICE quality standard on anxiety disorders [8]. CAMHS is commissioned to treat anxiety disorders in its model service specification [5]. Its reference list of anxiety disorders (e.g., generalized anxiety disorder, social anxiety disorder, etc.) however does not include phobias, despite their being categorized as an anxiety disorder in the NICE quality standard document. Some providers have used this omission as a mechanism for not offering treatment to this group. Delineating clear treatment options (as well as updating NICE guidelines) in the various treatment settings for specific phobia would likely address the inconsistency in service provision for phobia, and other mental health conditions, and provide a clear model of care in which new interventions can be embedded.

Better integration of mental health services for CYP, both within and between services, is also required, including an improved understanding of who should be doing what in any locality. Clear information about available care pathways for CYP and how they can be accessed is also needed and should be disseminated broadly. For research (and treatment) of specific phobias within CAMHS to be effective, we need more clarity from services providing specific phobia care around acceptance thresholds, and referral pathways.

When designing a trial it is important to review the relevant care pathways across a range of services, preferably across different regions and types of services, to try to capture the potential differences that may arise. Where there are a range of services, considering how the sites will be funded is important. In NIHR-funded research trials, treatment is funded by the usual NHS commissioning [9], but the same level of funding may not be available from other organizations.

### Challenge 2. Identifying the “real” service gatekeepers

Another key challenge the ASPECT team faced was identifying influential gatekeepers at all levels of recruiting sites that can facilitate promotion and delivery of the research. For example, staff working in Research and Development (R&D) within NHS Trusts are essential to disseminating the study to clinical service managers, who in turn promote the study to the clinical staff and administrative teams that often deliver the research on the ground. When embedding ASPECT within services, a disconnect frequently occurred between those making decisions further up the hierarchy (e.g., service managers), and those actually delivering the trial. This had implications for both the initial buy-in to ASPECT and subsequent site performance. For example, we regularly experienced occasions where site participation was agreed by those in managerial positions within the Trust; however, when ASPECT opened, the clinical and administrative staff on the ground were unable to deliver the trial effectively. This was typically due to high caseloads precluding trial engagement, an insufficient number of CYP phobia referrals, and lack of experience with research procedures in general (discussed further later). This disconnect between what might be achievable from a managerial perspective, and what was achievable on the ground commonly led to underperformance against pre-specified recruitment targets. Moreover, we experienced difficulties identifying gatekeepers within NHS Trust R&D teams. When attempting to expand the number of study sites, 22 Trusts (out of 77 we approached; 28.5%) progressed no further than contact with the R&D department who were either non-responsive or stated the study would not be suitable within their trust (and it was often unclear whether ASPECT had been presented to local clinical services at all).

#### Possible solutions

To effectively engage with services it is important to involve all levels of the service early on. We ensured representation from all levels of the service hierarchy (e.g., R&D manager, clinical service operational manager, clinical director and therapists) in initial discussions about ASPECT's suitability within their service. This approach, although more resource intensive, allows for the identification of possible barriers to trial implementation from all perspectives within services at an earlier stage, and facilitates collaborative solutions from the outset. For example, in one well-recruiting site the research team presented at a conference attended by a wide variety of professionals within the service. The research team shared information about ASPECT and facilitated a discussion about how the trial would work in practice. They offered reassurance about the support they could provide the clinical team during trial participation and were able to address and troubleshoot any concerns. By including professionals from multiple positions within a service it is possible to confirm that there is both front-line capacity to run the research, and that this has managerial support.

### Challenge 3. Identifying appropriate practitioners to deliver interventions

Embedding research into sites requires the identification of appropriate practitioners to deliver the interventions under investigation. However, when delivering pragmatic research into CAMHS settings comprising varied care pathways, this can be challenging. For ASPECT this included identifying individuals to deliver CBT-based interventions and those willing to be trained in OST. Given that NICE guidelines stipulate CBT for anxiety disorders in CYP, ASPECT's intervention delivery model at the trial's inception was largely predicated on the commissioned model where trained CBT therapists delivered interventions. However, as discussed, structural changes and reduced funding within CAMHS reduced staff numbers and specific phobias were deemed of lower priority than some other mental health difficulties. Therefore, many of the services identified for participation in ASPECT were not providing CBT or were referring phobia cases to alternative providers and using lower intensity non-CBT based services. As a result, the practitioners available to deliver ASPECT varied greatly in their roles and experience, ranging from school wellbeing workers to consultant psychiatrists. Such differences can have implications for intervention delivery, with those less experienced requiring additional support and those more experienced at risk of contaminating the findings by adapting the intervention delivery (e.g., by adding additional treatment components).

Furthermore, where referrals were passed to alternative providers, those services often felt ill-equipped to provide the research interventions. Therefore, it was necessary for the trial team to provide substantial training and support to services for the implementation of ASPECT's treatments. In some instances therapists expressed that they still lacked confidence in delivering the intervention following the training, and this was exacerbated if a delay occurred between training and intervention delivery. As part of ASPECT a “train the trainer” approach was also introduced. The aim of this was for more experienced therapists to be able to train and supervise more junior therapists in their service to deliver OST.

#### High staff turnover

Further compounding the challenge of identifying suitable staff to deliver research interventions within CAMHS is the high staff turnover within these settings. In ASPECT, this was a continual challenge with therapists we had originally trained in the trial processes and interventions leaving or assuming different roles. This loss of knowledgeable and enthusiastic staff often then reduced the sites' ability to recruit and deliver the research interventions. We hoped that the adoption of the “train the trainer” approach mentioned above might mitigate the impact of high staff turnover, however we found the “trainers” also left services or found they did not have the time to dedicate to training others.

#### Possible solutions

To implement research successfully, we recommend that appropriate therapists for intervention delivery are identified at the outset and that considerable guidance and training is available, and that this is appropriately costed. It is important to ensure that intervention training accounts for staff diversity and is developed to be accessible and inclusive to varying levels of expertise. This may include lengthening training sessions for those with less experience and providing additional supervision where required. To capitalize on therapist enthusiasm and facilitate a successful transition from intervention training to delivery, we encouraged therapists to identify a potential phobia case to bring to training sessions. This allowed a more tailored approach and helped trainees to be more confident from the off. In addition, we recommend having contingency plans in place to address staff turnover for the duration of the research. We initiated a rolling training programme to new CAMHS staff throughout trial recruitment, but this proved time intensive and expensive. Being cognizant of the changing capacity of services to successfully deliver research interventions means that training delivery should be planned and costed throughout the trial and not just at the beginning.

### Challenge 4. Limited service capacity and knowledge to enact research processes and practices independently

Besides delivering interventions, many research studies require participating sites to enact additional processes and practices to meet research objectives, including adherence to trial protocols and the completion of study documentation. Busy therapists may struggle to make time for these time-intensive additional tasks. Where services can allocate time for these activities, there is not necessarily the knowledge and experience of research to successfully enact them. This was evident in ASPECT where adherence to completing important trial documentation varied greatly across sites.

#### Possible solutions

To aid the enactment of research in CAMHS, we would recommend that research knowledge and training should be increased where it is limited. From an organizational perspective this could be encouraged through structural CYP mental health service initiatives such as training ‘Research Champions' who have allocated time to learn more about research processes and methodologies and/or research-based placements/secondments for CYP mental health service staff and trainees. This approach would aid future research delivery; enhancing the representation of under-served groups and preventing burdening the most experienced research sites. However, this requires time, money, and institutional buy-in from CAMHS and the NIHR Comprehensive Research Network. In the short-term, research teams working with CAMHS may need to consider prioritizing sites that have staff with a research background, though this should be considered alongside broad geographical and socioeconomic representation of the trial sample. In our experience, we saw more success where external collaborators with research experience worked closely with CAMHS on ASPECT (e.g., Research Nurses and local R&D teams) and where sites (e.g., voluntary sector) were well supported.

### Challenge 5. The impact of long waiting times on trial procedures

A final challenge we encountered during ASPECT was the variability in waiting times between services. Across the UK, the median quoted waiting time to begin treatment is around 2 months, although this only includes those who have had two contacts and not CYP who have been redirected elsewhere, making the true figure uncertain. Some NHS Trusts, including several who participated in ASPECT, have much longer times to treatment up to a median of 182 days [10]. The ASPECT protocol dictated that after a participant was randomized, they needed to have received therapy within 6-months before the final follow-up outcome measures were assessed. This 6-month limit combined with long-waiting times meant that, in several instances, CYP went through the trial without having started therapy, or in some cases, without having had an initial CAMHS assessment appointment.

#### Possible solutions

The ASPECT team worked with services, where possible, to adapt the research to the waiting times in place, for example, by delaying randomizing participants (and therefore starting the 6-month clock) until they were near the top of the waiting list. To implement this, in some services, those referring young people would monitor their progress on the waiting list and make the referral to ASPECT when their initial appointment was imminent. Although this required commitment of additional time and resources from both the trial team and various site staff it enabled more CYP to receive treatment before follow-up.

We advise researchers to consider the long waiting lists (and variation across NHS Trusts) when designing future studies and explore whether this would differ between the trial arms. The timing of randomization relative to these waiting lists is also an important consideration. Furthermore, we recommend researchers take an individualized approach with sites and work with them to understand how the research, and any associated processes, would work most effectively within their services and timetables. Researchers need to be adaptable to service processes and requirements, ensuring that they work alongside services to provide a positive and supportive research experience that will enhance motivation to participate in future research. The challenges encountered during ASPECT as well as recommendations to mitigate them are summarised in Table 1.

**Table 1:** A summary of the challenges encountered during ASPECT and possible solutions to mitigate these.

**Challenge**	**Possible solutions**
Service delivery restructuring and lack of streamlined care pathways	• Readily available and updated NICE guidelines • Delineating clear treatment options • Better integration of mental health services for CYP • Clear information about available care pathways
Identifying the ‘real' service gatekeepers	• Involvement of individuals from all positions within the service hierarchy in initial discussions • Collaborative approach in identifying barriers and troubleshooting
Identifying appropriate practitioners to deliver interventions	• Incorporate practical examples into training • Adopt contingency plans to address high staff turnover (e.g., rolling training programme) • Ensure training is accessible and inclusive to varied levels of expertise. • Prepare for increased training provision and ongoing support.
Limited service capacity and experience to enact research processes and practices independently	• Provide support with logistical matters • Training of research champions • Embed research within services with previous research experience • Identify external collaborators to support research delivery within CAMHS
The impact of long waiting times on trial procedures	• Consideration of waiting time variations across NHS Trusts and other services • Adaptation of research processes to waiting times e.g., the timing of randomization • Collaboration with services to effectively embed research within services • Adopting an adaptable and flexible approach to provide a positive and supportive research experience

## Discussion

This paper has explored some of the challenges to delivering large scale clinical trials in CAMHS, in terms of identifying sites, embedding research processes and delivering interventions into often already overstretched services, with ASPECT as a case in point. Many of these challenges also apply to the embedding of research findings into services when a trial has concluded. Despite the challenges, ASPECT was successful in meeting its recruitment, delivery and follow-up targets by using mitigation strategies. We hope that these strategies are useful for future researchers who wish to mitigate the challenges they face while undertaking research in CAMHS.

## Data availability statement

Any requests for sharing of data can be made to the corresponding author or Sheffield Clinical Trials Unit (ctru@sheffield.ac.uk). The ASPECT management team will consider the sharing of data on a case-by case basis in line with the ethical approval and patient information sheets.

## Ethics statement

The studies involving human participants were reviewed and approved by ASPECT received Health Research Authority and Research Ethics Committee approval from North East–York Ethics Research Committee [17/NE/0012] in February 2017. Written informed consent to participate in this study was provided by the participants' legal guardian/next of kin.

## Author contributions

BW, CC, KB, LG, RH, and JW conceived the study idea and designed the project. BW and LG also provided intervention training to clinicians. LT and AS managed the trial. EH was involved in the acquisition of the data. All authors contributed to writing this manuscript and have all read and approved the final version.

## Funding

Funding for this study was provided by the NIHR Health Technology Assessment programme (HTA15/38/04).

## Conflict of interest

The authors declare that the research was conducted in the absence of any commercial or financial relationships that could be construed as a potential conflict of interest.

## Publisher's note

All claims expressed in this article are solely those of the authors and do not necessarily represent those of their affiliated organizations, or those of the publisher, the editors and the reviewers. Any product that may be evaluated in this article, or claim that may be made by its manufacturer, is not guaranteed or endorsed by the publisher.

## Author disclaimer

The views and opinions expressed are those of the authors and do not necessarily reflect those of the NHS, the NIHR or the Department of Health and Social Care.

## Abbreviations

ASPECT: Alleviating Specific Phobia Experienced by Children Trial
CAMHS: Child and Adolescent Mental Health Services
CBT: Cognitive Behavioral Therapy
CYP: Children and Young People
NHS: National Health Service
NICE: National Institute for Health and Care Excellence
NIHR: National Institute for Health and Care Research
OST: One Session Treatment
R&D: Research and Development
SPA: Single Point of Access.
